# Use of lecithin to control fiber morphology in electrospun poly (ɛ‐caprolactone) scaffolds for improved tissue engineering applications

**DOI:** 10.1002/jbm.a.36139

**Published:** 2017-07-18

**Authors:** Benjamin D.M. Coverdale, Julie E. Gough, William W. Sampson, Judith A. Hoyland

**Affiliations:** ^1^ School of Materials The University of Manchester Manchester M13 9PL United Kingdom; ^2^ The Stopford Building School of Biological Sciences, Division of Cell Matrix and Regenerative Medicine, The University of Manchester Manchester M13 9TP United Kingdom; ^3^ NIHR Manchester Musculoskeletal Biomedical Research Unit, Central Manchester NHS Foundation Trust, Manchester Academic Health Science Centre Manchester United Kingdom

**Keywords:** electrospinning, tissue engineering, adhesion, scaffolds, pore size

## Abstract

We elucidate the effects of incorporating surfactants into electrospun poly (ɛ‐caprolactone) (PCL) scaffolds on network homogeneity, cellular adherence and osteogenic differentiation. Lecithin was added with a range of concentrations to PCL solutions, which were electrospun to yield functionalized scaffolds. Addition of lecithin yielded a dose‐dependent reduction in scaffold hydrophobicity, whilst reducing fiber width and hence increasing specific surface area. These changes in scaffold morphology were associated with increased cellular attachment of Saos‐2 osteoblasts 3‐h postseeding. Furthermore, cells on scaffolds showed comparable proliferation over 14 days of incubation to TCP controls. Through model‐based interpretation of image analysis combined with gravimetric estimates of porosity, lecithin is shown to reduce scaffold porosity and mean pore size. Additionally, lecithin incorporation is found to reduce fiber curvature, resulting in increased scaffold specific elastic modulus. Low concentrations of lecithin were found to induce upregulation of several genes associated with osteogenesis in primary mesenchymal stem cells. The results demonstrate that functionalization of electrospun PCL scaffolds with lecithin can increase the biocompatibility and regenerative potential of these networks for bone tissue engineering applications. © 2017 The Authors Journal of Biomedical Materials Research Part A Published by Wiley Periodicals, Inc. J Biomed Mater Res Part A: 105A: 2865–2874, 2017.

## INTRODUCTION

Electrospinning is an increasingly popular method of generating scaffolds for use in the field of regenerative medicine. It is inexpensive and can deliver highly porous structures with high surface area to volume ratios, which mimic the extracellular matrix (ECM). Poly (ɛ‐caprolactone) (PCL) is a Food and Drug Administration (FDA) approved semicrystalline polyester commonly used in regenerative applications. It has a compressive strength similar to that of trabecular bone^1^ and biodegrades slowly over the course of about 2 years.^2^ PCL scaffolds can support the proliferation and differentiation of a wide variety of cell types, including bone marrow derived mesenchymal stem cells.^3^ However, these scaffolds are typically hydrophobic, a characteristic that has been linked with poor biocompatibility, bioactivity, cellular attachment and scaffold invasion, thus limiting their use in regenerative applications.^4^, ^5^

Several methods have been employed to enhance the characteristics of hydrophobic polymers, including plasma treatment, surface modification and polymer coating.^1^, ^6^, ^7^ However, although immediately beneficial, these do not address the issue that PCL is biodegradable. Once the surface modification has been removed or exhausted, or as the scaffold begins to degrade, the associated benefits are lost as the hydrophobic surface is exposed. Incorporating factors into the electrospinning solution is one method of ensuring consistent modification throughout fibers. Gelatin and nanoclays have been used to this end, delivering a sustained increase in proliferation and adhesion of cells throughout the degradation of fibers.^8^, ^9^ Whereas these scaffolds offer significant benefits over surface treatments, they generally lack osteogenic characteristics, but nonetheless offer a more favourable method of scaffold fabrication for regenerative applications.

Electrospinning is a stochastic process, producing a distribution of fiber diameters and pore sizes, which can act to influence the growth and regulation of stem cells. Fiber, and hence scaffold morphology can be influenced, and controlled by varying parameters such as flow rate, applied voltage and collecting distance.^10^ Increased fiber diameter has been shown to reduce the specific surface area of scaffolds, which in turn reduces the number of attachment sites for cells.^11^ Reducing fiber diameter provides a greater surface area for attachment, but prevents cellular infiltration into scaffolds.^12^ Given the stochastic structure of electrospun scaffolds, it is important to exercise control over fiber parameters where possible in order to influence network structure; for example, design equations for pore size and specific surface area reveal a strong and coupled dependence of these properties on fiber diameter,^13^ so control of this variable is important. It follows that such control over fiber morphology will allow for tailored scaffolds, optimized for cellular attachment, proliferation, invasion, and stem cell differentiation.

This study aims to modify the morphologies of electrospun fibers and hence the structure of scaffolds through the incorporation of a naturally occurring surfactant, lecithin. Lecithin is a zwitterionic phospholipid that lowers the surface tension between two phases. We hypothesize that lecithin can be used to reduce hydrophobicity of electrospun PCL fibers, yielding more favourable surfaces for cellular attachment. It is known that the in‐plane mass uniformity of random fibrous networks increases with reduced fiber diameter;^14^, ^15^ we therefore hypothesize also, that reducing the surface tension of the solution will reduce variability in fiber diameters and hence yield more uniform scaffolds through the reduced incidence of larger diameter fibers. The production of structures closer to native ECM could lead to electrospun scaffolds better suited to regenerative applications, such as a replacement for bone grafting.

## MATERIALS AND METHODS

### Electrospinning of nanofibrous substrates

A 10% w/v solution of PCL (Sigma‐Aldrich) was produced by dissolving PCL pellets in 1,1,1,3,3,3‐Hexafluoro‐2‐propanol (HFIP; Alfa Aesar). Powdered lecithin (VWR) was mixed into the solution via continuous magnetic stirring at room temperature. For 20% lecithin scaffolds, 0.2 g lecithin was added per 10 mL 10% PCL solution. This concentrated solution was subsequently diluted to produce scaffolds containing 5, 2, and 1% lecithin. Using a 10 mL syringe, solutions were electrospun through a copper electrode from a blunted 21‐gauge needle at a rate of 2.5 mL/h (applied voltage at the needle 12 kV and mandrel potential −5 kV). The spinning distance was 20 cm and fibers were spun for 90 min for all samples.

### X‐ray photoelectron spectroscopy

Electrospun scaffolds were cut into 1 cm^2^ segments and mounted onto the XPS AXIS Ultra recording instrument with double‐sided tape. High resolution scans were undertaken for P, N, C, O, and F elements overnight and analysed using CasaXPS software.

### Water contact angle

Scaffolds were cut into strips 5 cm in length and mounted onto glass slides with double sided tape. Tissue culture plastic (TCP) was used as a control. Contact angles were measured using a Krüss Drop Shape Analyser 100 platform. The apparatus was set to dispense 21 µL drops of distilled H_2_O whilst recording the process at 30 frames per second. Images immediately following the water drop impact were used for analysis. Sessile drop algorithms were used to calculate the water contact angle.

### Mechanical testing

Tensile testing was performed using an Instron 3344 Single Column System fitted with a 100*N* static load cell. Testing was carried out at a crosshead speed of 25 mm/min with an initial jaw separation of 30 mm. Scaffolds were cut into rectangles measuring 40 mm × 5 mm using a disposable scalpel. This allowed 5 mm either end of the scaffold for anchorage into clamps. Specific elastic modulus (N m g^−1^) was determined as the maximum gradient of the initial linear region of a plot of load per unit width against strain, divided by the network areal density (g m^−2^). All results presented are the average of six repeats. For nanoindentation, solvent cast films were prepared through spin coating. 13 mm coverslips were locked on to the spin coater via vacuum. Two drops of polymer solution were placed in the middle of the coverslip with a Pasteur pipette, before spinning for 40 s at 5000 rpm. Films were secured onto a nanoindenting block via superglue and analysed using an MTS Nanoindenter XP using NanoSuite software. 20 indentations were made per sample at a depth of 200 nm. Drift rate was set to 0.15 nm/s and approach velocity was 10 nm/s. Data was expressed as a function of modulus at maximum load.

### Fiber diameter and pore size analysis

Samples were mounted onto aluminium stubs with carbon tabs and gold coated using an Edwards S150B sputter coater. SEM images were taken using a Phenom G2 Pro SEM coupled with the Phenom Fibermetric analysis package. Five images were taken per sample at a field size of 100 µm at a resolution of 0.1 µm/pixel. The diameters of 100 fibers were measured from each image and data exported for analysis. Pore areas were determined by manipulating image colour thresholds in ImageJ to achieve a single layer of fibers. Pores areas were measured; pores lying at the image edge were excluded, as were those smaller than 5 µm^2^. Following Eichhorn and Sampson,^13^ equivalent pore diameters, *d*, were calculated as the diameter of a circle with the same area as a pore, *a*:

(1) $$d=2\sqrt{a/\pi }$$

Densities of scaffolds, *ρ*_s_, were estimated by weighing samples of known area to determine their areal density and dividing this by thickness of scaffolds. Porosities of scaffolds were obtained through comparison of the density of scaffolds, *ρ*_s_, with that of the solid polymer density, *ρ*_p_:

(2) $$ɛ=1-{(\rho }_{s}/\rho_{p})$$

### Cell culture of Saos‐2 osteoblasts

Saos‐2 osteoblasts (Sigma‐Aldrich) were cultured with McCoy's 5 A modified medium supplemented with 10% fetal bovine serum (FBS), antibiotic solution (100 mg/mL streptomycin, 100 U/mL penicillin) and 2 mM l‐glutamine. Cells were cultured until confluent at 37°C, 5% CO_2_.

### Cell culture of primary bone marrow derived MSCs

Primary MSCs were cultured with Dulbecco's Modified Eagle Medium (DMEM) supplemented with 10% FBS and antibiotic solution (100 mg/mL streptomycin, 100 U/mL penicillin). Cells were cultured until confluent at 37°C, 5% CO_2_.

### Cellular attachment and proliferation

Saos‐2 osteoblasts were seeded at a density of 60,000 cells per scaffold in low attachment 24‐well plates (Corning; 1.9 cm^2^) and incubated for until the desired time point. Media was replaced before 100 µL of AlamarBlue solution (Sigma Aldrich) was added. Following a 2‐h incubation period (37°C), 200 µL of solution was transferred to black 96‐well plates in triplicate. Fluorescence was measured using a FluoStar Optimax Spectrofluorometer with an excitation wavelength of 530 nm and an emission wavelength of 590 nm. Cell numbers were calculated by converting fluorescence values using corresponding standard curves.

### *In vitro* differentiation

Primary bone marrow derived MSCs were seeded at a density of 60,000 cells per scaffold. Total RNA was harvested at day 21 using TRIzol reagent (Sigma Aldrich). RNA was quantified using a Nanodrop (Thermo Scientific). RNA was reverse transcribed and gene expression analysed quantitatively using Sigma mastermix with primers and FAM‐BHQ1 probes, with a StepOnePlus Real‐Time PCR System (ThermoFisher Scientific). Data were analyzed using the 2^–ΔΔ^ Ct method, normalizing expression to two housekeeping genes (MRPL19 and GAPDH).^16^ Cells cultured on TCP for 21 days were used as controls.

### Statistical analysis

Statistical analysis in all cases was performed using GraphPad Prism 7.0 software. Data were assessed with one‐way analysis of variance (ANOVA) and Turkey *post hoc* test for comparison of groups. *p* values < 0.05 were considered statistically significant. Data are expressed as means ± one standard deviation.

## RESULTS

### Confirmation of lecithin presence with XPS

**Figure 1:**
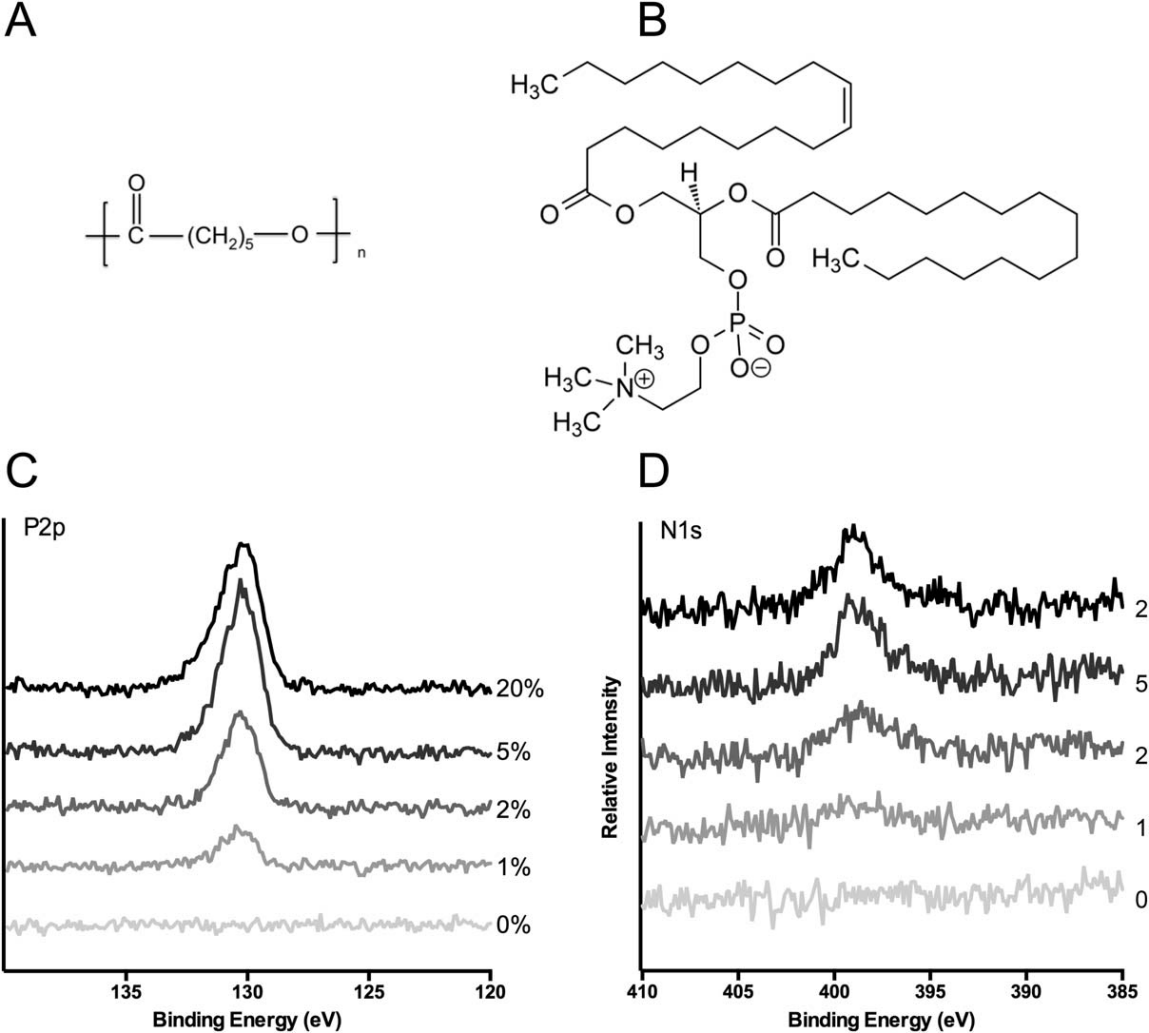
XPS analysis of electrospun scaffolds containing lecithin. A: Chemical structure of PCL. B: Chemical structure of lecithin. C,D: High resolution XPS scans showing relative intensities of phosphorous (C) and nitrogen (D) on PCL scaffolds containing 1, 2, 5, and 20% lecithin.

Observation of the chemical structures of PCL and lecithin [Fig. 1(A,B)] reveals key differences in their chemical composition. PCL contains only carbon, hydrogen and oxygen, whilst lecithin additionally contains a small amount of phosphorous and nitrogen. Phosphorous and nitrogen residues are clearly evident in all lecithin‐containing samples, indicating the surfactant has been successfully incorporated into electrospun fibers [Fig. 1(C,D)]. Importantly, the high‐resolution spectra for both phosphorous and nitrogen show an increase in peak intensity and breadth, corresponding with increasing lecithin concentration. Theoretical compositions of both phosphorous and nitrogen, detailed in Table 1, were similar to values determined through XPS analysis.

**Table 1:** Theoretical and Actual Composition of Phosphorus and Nitrogen in PCL Scaffolds Containing 0, 2, and 20% Lecithin

	Theoretical Composition		Actual Composition	
	*P* %	*N* %	*p* %	*N* %
0%	0.00	0.00	0.00	0.03
2%	0.22	0.22	0.46	0.47
20%	1.09	1.09	0.97	0.78

### Water contact angle

**Figure 2:**
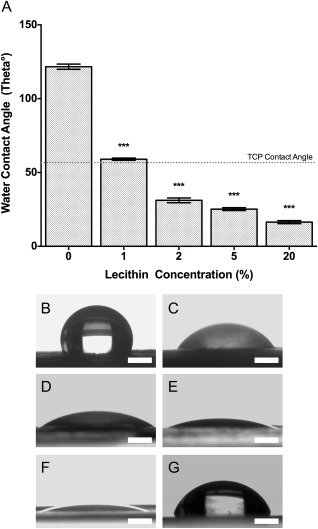
Water contact angle analysis. A: Bar graph showing water contact angle from a 20 µL drop of dH_2_O on lecithin containing scaffolds. TCP control is marked by the dotted line. B–G: Images of water droplets immediately following droplet impact on PCL, 1, 2, 5, 20, and TCP. *n* = 10. ***p < 0.001. All values compared to PCL scaffolds. Scale bar 1 mm.

PCL scaffolds were the most hydrophobic producing a contact angle of 121.7° [Fig. 2(A,B)]. Conversely, lecithin‐containing scaffolds had significantly reduced contact angles. 1% lecithin was found to reduce the contact angle to 59.0° whilst the highest concentration of lecithin, 20%, produced the smallest contact angle of 16.4° [Fig. 2(A,F)] illustrating that lecithin acts in a dose dependent manner on the hydrophobicity of PCL scaffolds. In all lecithin‐containing scaffolds, the water droplet was completely absorbed moments after impact, highlighting increased hydrophilicity of the modified scaffolds. TCP produced a significantly reduced contact angle compared to PCL (56.4°).

### Mechanical properties

**Figure 3:**
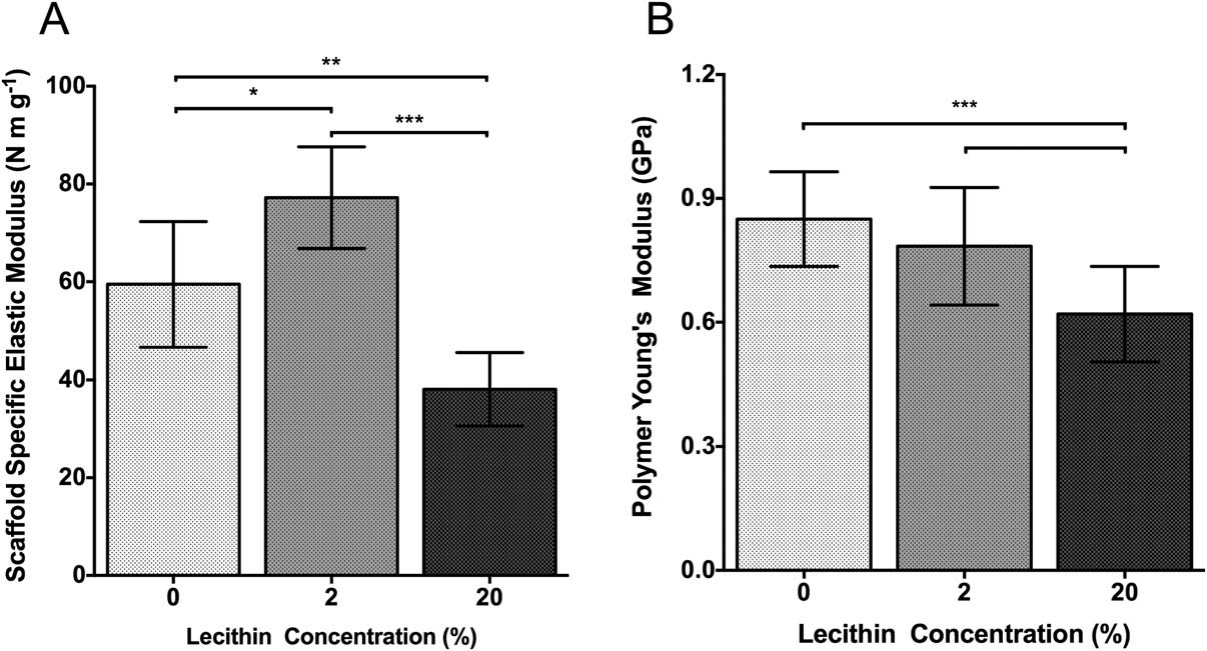
Mechanical properties of scaffolds containing lecithin. A: Scaffold specific elastic modulus of electrospun scaffolds. *n* = 6. B: Polymer Young's modulus of spin cast films, *n* = 20. ***p < 0.001.

Addition of 2% lecithin to the PCL resulted in an increase of around 30% in specific elastic modulus. Importantly, increasing the lecithin content to 20% yielded a significantly reduced specific elastic modulus compared to both pure PCL and 2% lecithin scaffolds [Fig. 3(A)].

The modulus of the polymer was carried out via nanoindentation of spin cast films mounted on glass slides. No significant difference was observed between PCL films and those containing 2% lecithin, although the Young's modulus of films containing 20% lecithin is markedly lower [Fig. 3(B)]. We expect these properties of films to correspond to those of the constituent fibers of electrospun scaffolds; the effects on the modulus of scaffolds, as shown in Figure 3 are the subject of our subsequent discussion.

### Fibermetrics and pore diameters

**Figure 4:**
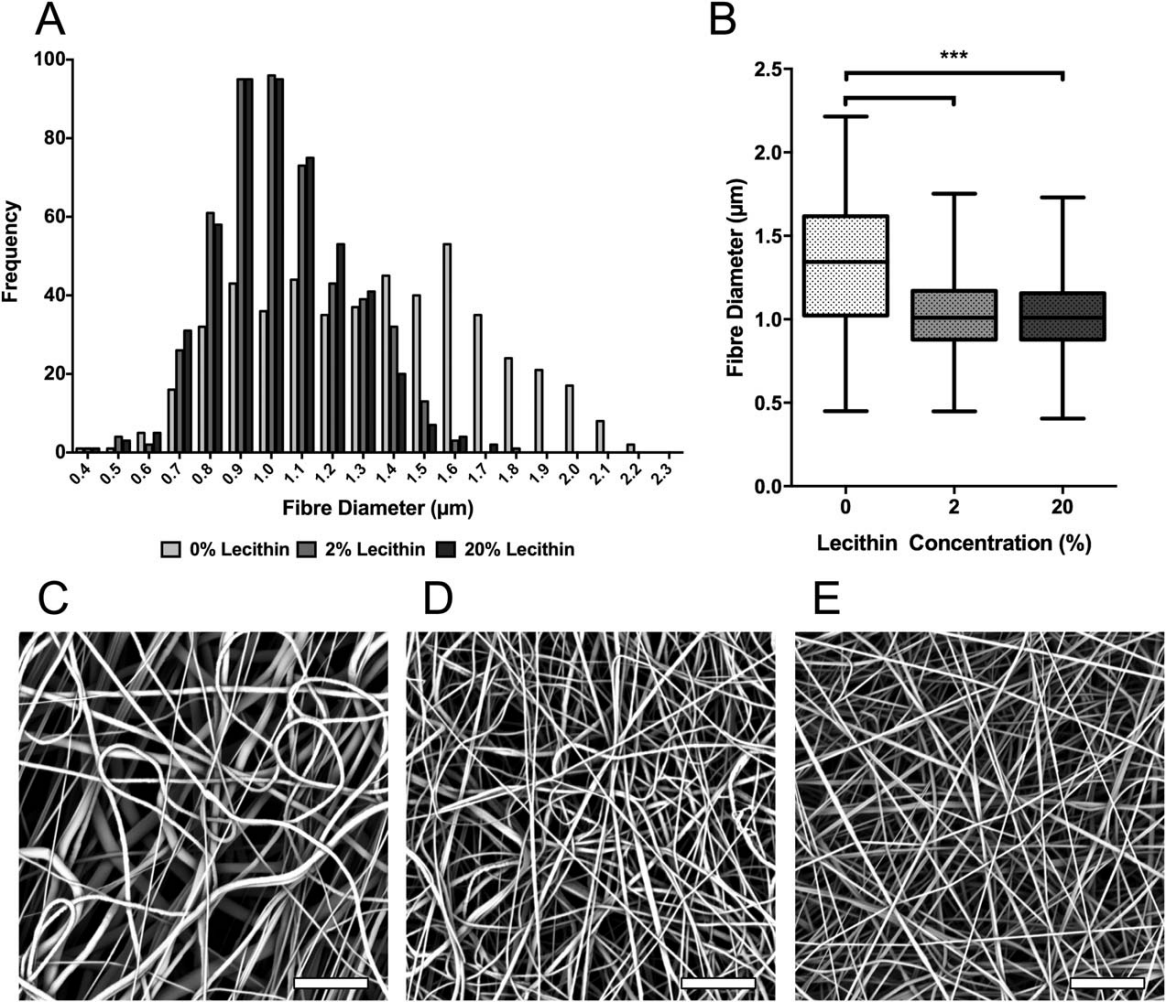
Electrospun fiber diameter analysis. A: Histogram of fiber diameter distribution comparing PCL 2 and 20% lecithin scaffolds. B: Box and whisker plot of fiber diameters showing maximum, minimum, mean and interquartile range of PCL 2 and 20% lecithin scaffolds. C–E: SEM of PCL scaffolds containing 0, 2, and 20% lecithin. ***p < 0.001. Scale bar 20 µm.

PCL scaffolds show a distribution of fiber diameters ranging from 0.4 to 2.2 µm with a mean diameter of 1.3 µm [Fig. 4(A,B)]. The majority of fibers also exhibit significant curvature. Upon the addition of both 2 and 20% lecithin to PCL, this fiber diameter distribution was reduced, to a minimum size of 0.4 µm and a maximum size of 1.8 µm, with an additional reduction in mean fiber diameter to 1.0 µm [Fig. 4(A,B)]. Importantly, increasing lecithin concentration also reduced the curvature of fibers [Fig. 4(D)]. Despite the reductions in diameter distribution and mean fiber diameter, the coefficient of variation of fibers within these scaffolds was similar: 21% for PCL scaffolds and 18% for scaffolds containing 20% lecithin. Thus, although the addition of lecithin reduces fiber diameters, the range of observed values is similar, when normalized by the mean. The reduction in mean fiber diameter caused by the addition of lecithin was calculated to reduce the specific surface area of electrospun scaffolds. Following Eichhorn and Sampson, specific surface area was calculated using Eq. (3), where *ρ*_p_ is the density of the polymer (kg m^−3^) and *ω*_0_ is mean fiber diameter (µm).

(3) $$S_{f}=\frac{4}{\left(\rho_{p}\omega_{0}\right)}$$

Scaffolds with no lecithin, with a mean diameter of 1.3 µm were found to have a specific surface area of 2630 m^2^/kg whereas 20% lecithin scaffolds with a mean diameter of 1.0 µm presented more available specific surface area, 3500 m^2^/kg.

**Figure 5:**
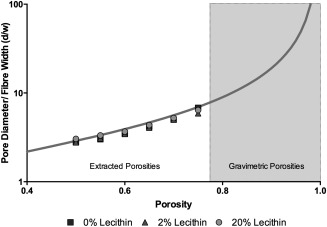
Scaffold fibermetrics. Mean pore dimensions normalized to fiber diameter plotted against porosity, as varied by image analysis.

From statistical considerations, Eichhorn and Sampson^15^ show that the mean equivalent diameter, $\bar{d}$, is related to the fiber diameter, ω, and network porosity, *ɛ*, by:

(4) $$\bar{d}=\frac{2\omega }{\log(1/ɛ)}$$

such that, in a network of given porosity, the pore dimension is directly proportional to the width of fibers. From inspection of the micrographs in Figure 4, it is evident that fibers partition the network into voids through the depth of the scaffold making it difficult to directly measure void dimensions in any specific plane. Pore areas were measured from micrographs using the image‐analysis software, ImageJ. In order to measure pore areas, it was necessary to apply a threshold to separate fibers from voids. From gravimetric analysis, we estimate the porosity of the PCL network to be of order 0.85; when a threshold was applied to a micrograph such that this fraction of the image represented voids, it was difficult to identify individual voids to the contribution of fibers in different layers. Accordingly, we chose instead to measure void areas at a range of threshold values such that different values were obtained from the image corresponding to different porosities. From these pore areas, equivalent pore diameters were calculated using Eq. (1). The resultant data are plotted in Figure 5, along with the predicted relationship given by Eq. (4) and represented as a solid line. Note that pore diameters have been normalized by fiber width, aiding comparison for our scaffolds with different fiber width. Porosities of <0.5 have not been included in our analysis, since these are not representative of the range seen in electrospun networks. Instead, for cases where the image is overwhelmingly voids (i.e., *ɛ* > 0.5) pore sizes have been extracted and pore diameters calculated. Measurements for PCL scaffolds at porosities >0.5 follow the model very closely (Fig. 5) confirming that pore size in our scaffolds is directly related to fiber diameter. The addition of lecithin has no effect on this relationship. Data for both 2 and 20% lecithin scaffolds overlay those of PCL, indicating that even with the addition of lecithin the relationship between pore size and fiber diameter is unaffected (Fig. 5), as predicted by Eq. (4). For porosities >0.75, thresholding yielded interconnected voids that did not permit meaningful measurement of pore diameters. However, given our confidence in the predictions of Eq. (4) arising from the analysis presented here, theoretical values for pore diameters of each scaffold have been calculated using scaffold porosities determined gravimetrically (Table 2). Additionally it is important to consider pore diameters have been calculated through in‐plane pore dimensions. In fibrous networks such as these, pore height, in the perpendicular plane, is expected to be at most half that of the in‐plane pore diameter.

**Table 2:** Table Expressing Gravimetrically Calculated Porosities and Equivalent Pore Diameters as Determined by Eq. (4)

Scaffold	Fiber Diameter (µm)	Gravimetric Porosity (%)	Calculated Mean Pore Diameter (µm)
0%	1.3	86	17.0
2%	1.0	81	9.9
20%	1.0	79	8.8

### Cellular proliferation and attachment with Saos‐2 osteoblasts

**Figure 6:**
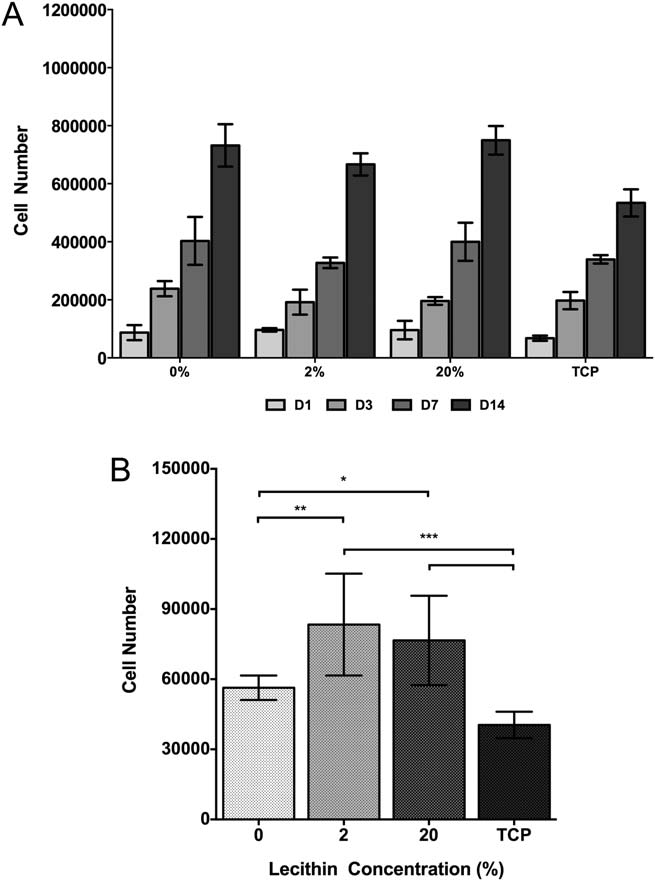
Cellular attachment and proliferation. A: Metabolic increase of Saos‐2 osteoblasts cultured on electrospun PCL scaffolds containing lecithin over 14 days of culture. All cell number increases for each concentration are significant at each time point. *n* = 9. (***p < 0.001). B: Initial cellular attachment of Saos‐2 osteoblasts on electrospun PCL scaffolds functionalized with lecithin after 3 h of incubation. *n* = 9. *p < 0.05, **p < 0.01, ***p < 0.001.

Following an initial seeding density of 60,000 cells per well, all scaffolds showed a significant increase in cell number at each time point up to 14 days in culture [Fig. 6(A)]. As expected, the control sample, TCP, exhibited increasing cell numbers at each time point. PCL as well as 2 and 20% lecithin scaffolds exhibited very similar trends in both growth rates and final cell numbers. Following 14 days, both PCL and 20% lecithin scaffolds had given rise to almost 750,000 cells per well, whilst 2% scaffolds had produced over 650,000 per well [Fig. 6(A)].

Cellular attachment was assessed to determine whether the increased hydrophilicity of lecithin containing scaffolds could be related to enhanced cellular adherence. The addition of lecithin stimulated increased cellular attachment compared to PCL scaffolds and TCP [Fig. 6(B)]. Both 2 and 20% lecithin scaffolds enabled the attachment of over 20,000 more cells than PCL scaffolds, and over 35,000 more than TCP (*p* < 0.05).

### Osteogenic effects of lecithin on PCL scaffolds with bmMSCs

**Figure 7:**
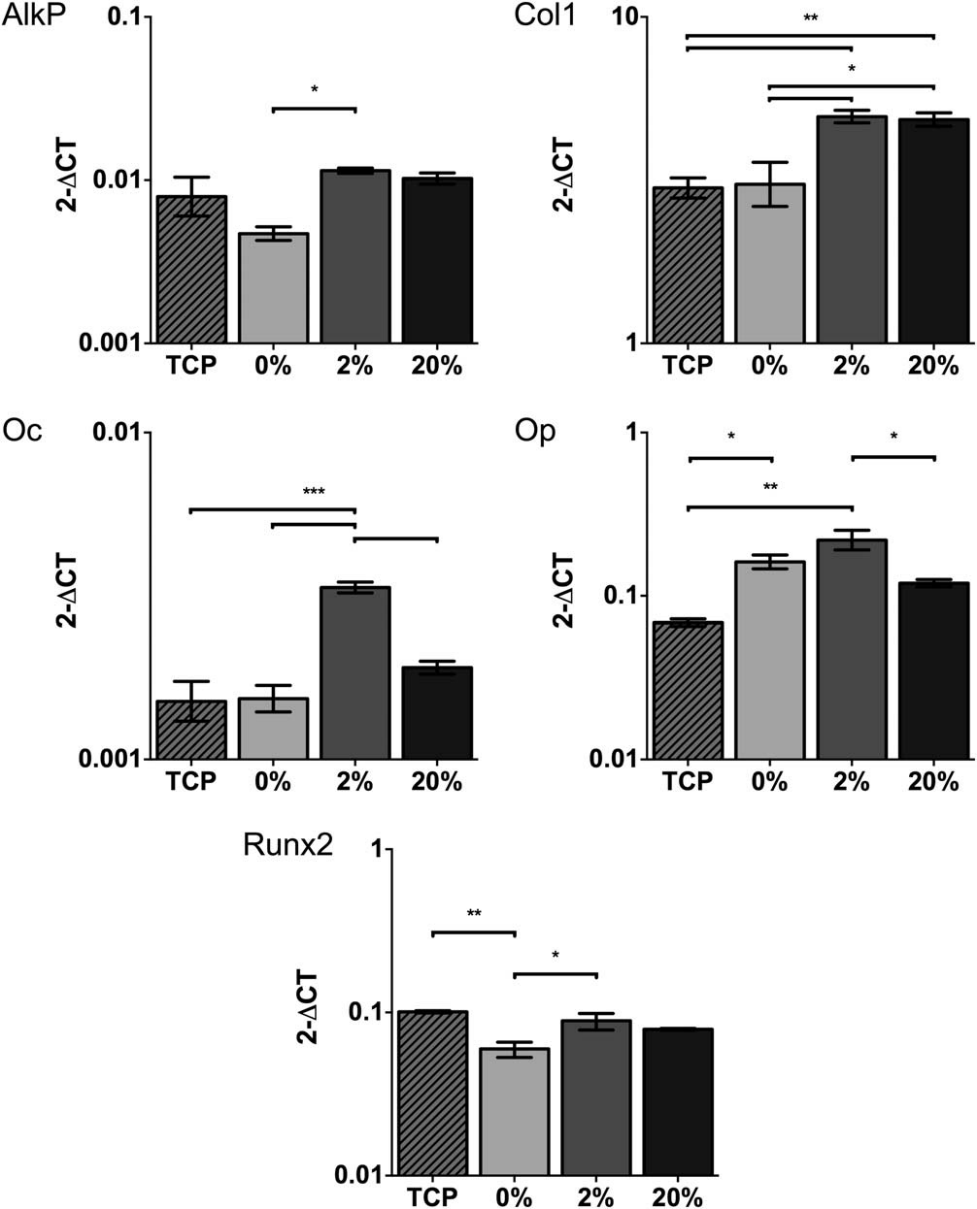
Quantitative real‐time PCR analysis of gene expression in response to scaffolds containing various concentrations of lecithin after 21 days of incubation. Relative gene expression was normalized to mean housekeeping genes (GAPDH and MRPL19) Cells cultured on tissue culture plastic for 21 days were used as controls. *n* = 3. *p < 0.05, **p < 0.01, ***p < 0.001.

Scaffolds functionalized with 2% lecithin gave rise to a significant upregulation of gene expression for alkaline phosphatase, collagen 1, osteocalcin and Runx2 when compared to PCL scaffolds. There was no significant difference in expression between PCL and 2% scaffolds for osteopontin, despite 2% scaffolds showing enhanced upregulation (Fig. 7). Conversely, high concentrations of lecithin generally did not elicit upregulation of osteogenic genes when compared to PCL scaffolds as no significant differences were observed. 20% scaffolds however did show a significant increase in expression of collagen 1.

## DISCUSSION

Reducing scaffold hydrophobicity has become paramount in order to create scaffolds that can support increased cellular proliferation and adhesion. Here, we show how lecithin, a naturally occurring phospholipid, was successfully incorporated into electrospun PCL fibers through its addition to the electrospinning solution. XPS analysis clearly shows a dose dependent increase in peak intensity for phosphorous and nitrogen residues as lecithin concentration is increased, confirming incorporation into the scaffold (Fig. 1). Compositions of phosphorous and nitrogen, recorded at the surface of the fibers, were similar to theoretical estimations indicating incorporation throughout the scaffold. If lecithin were localized to the core or shell of the fibers, the theoretical concentrations would have been respectively lower or higher than anticipated. Since this outcome was not observed, uniform incorporation must have occurred. This is especially important with biodegradable polymers, as modifications must be available throughout the lifetime of the scaffold. Surface alterations such as plasma treatment, ozone induced grafting and surface coatings provide temporary improvements to scaffolds,^1^, ^17^, ^18^ however they will be lost as the scaffold degrades.

Despite lecithin being incorporated into PCL scaffolds, surface effects could still clearly be seen following water contact angle analysis (Fig. 2). Lecithin acts in a dose‐dependent manner, reducing the contact angle of PCL fibers from 121.7° to 16.4° with the incorporation of 20% lecithin demonstrating that isolating factors to the surface of electrospun scaffolds is not advantageous over incorporation as the effects are still evident. Coating methods such as those shown by Bassi et al.^1^ deliver a single change in surface properties, but incorporation of factors, such as lecithin can provide a range of contact angles which can be tailored for a specific need.

The addition of 2% lecithin allowed for a significantly increased scaffold specific elastic modulus compared to PCL scaffolds [Fig. 3(A)]. On inspection of micrographs, it is clear that addition of lecithin greatly reduces fiber curvature. Intuitively, we expect that the first effect of applying a strain to a network of curved fibers, such as those in the pure PCL scaffold, is to straighten those fibers before they begin to actively bear load. In contrast, the straighter fibers of the networks containing 2% lecithin will bear load more rapidly, resulting in the higher specific elastic modulus of the scaffold, despite the polymer modulus being slightly lower [Fig. 3(A,B)]. Fibers are further straightened with the addition of 20% lecithin, so the reduction in scaffold specific elastic modulus for the networks containing 20% lecithin can be attributed to the reduction in polymer modulus. The results demonstrate that through careful addition of additives, the effects of polymer modulus and network modulus can be decoupled, permitting manufacture of structures with targeted mechanical properties.

Fiber diameter distribution was reduced with the addition of lecithin. PCL scaffolds have a larger mean fiber diameter (1.3 µm) than those containing lecithin, though the distribution of fiber diameter relative to the mean, as characterized by the coefficient of variation, is unaffected. It is understood that stem cells can respond to slight changes in surface topography^19^, ^20^ therefore producing scaffolds which are as regular as possible will allow for greater control of cellular proliferation, migration and most importantly differentiation of a variety of cell types. Other studies incorporating factors, such as nanoclay into scaffolds to enhance biocompatibility lack this regularity, becoming beaded and inconsistent with the additions.^9^

Reduction in fiber size also reduces pore diameter. Eichhorn and Sampson have shown that in a network of known porosity, mean pore size is directly proportional to mean fiber width.^13^ A reduction in fiber diameter will therefore also reduce mean pore size of the scaffold. Lecithin addition to PCL scaffolds clearly had no effect on the pore and fiber size relationship as can be seen from both the extracted and gravimetric porosities. All values lie close to the theoretical model, indicating as lecithin reduces fiber diameter, the pore sizes of the scaffold will be proportionally smaller. However although lecithin would appear to make scaffolds more uniform, this is not the case. Large fibers give rise to large pores, but both fiber diameter and pore size reduce relative to one another upon the addition of lecithin. Confirmed by the constant coefficients of variation of fiber diameters, lecithin acts to control scaling of the scaffolds instead of the regularity of fibers and pores.

It is known that a reduction in scaffold pore size restricts cellular ingression;^12^ however, this reduction leads to increased specific surface area of scaffolds providing more available sites for cellular attachment.^11^ In this study, 20% lecithin scaffolds produced a 33% increase in specific surface area compared to PCL scaffolds. This increased surface area combined with reduced hydrophobicity led to a significant increase in cellular attachment on lecithin containing scaffolds. After 3 h, both 2 and 20% lecithin scaffolds had significantly more attached cells than both PCL scaffolds and TCP. The addition of lecithin therefore dramatically improved the seeding efficiency of PCL. All scaffolds allowed sustained cell growth over 14 days of culture, however neither concentration of lecithin allowed for enhanced proliferation over PCL, as was suggested by the aforementioned attachment assay. Whilst increased surface area can augment initial attachment, scaffolds with large pore diameters have been shown to support increased cell growth over longer periods.^21^ Although PCL is hydrophobic, it is possible the larger pore diameters enabled proliferation into the scaffold, giving rise to higher than expected cell numbers.

Additionally, 2% lecithin scaffolds showed significant upregulation of expression for alkaline phosphatase, collagen 1, osteopontin, and Runx2 when compared with controls, indicating osteoinduction had occurred. Although 20% lecithin scaffolds elicited an increase in collagen 1 expression, no other increases were observed, suggesting high concentrations of lecithin restrict osteogenic differentiation. It is likely this observation is due to reduced scaffold stiffness^22^ since high concentrations of lecithin have been shown to hinder the mechanical properties of these networks. These results however indicate that in small doses, lecithin can transform electrospun PCL into an osteoinductive scaffold. Blending lecithin throughout the entire fiber, extends osteoinductive properties of the scaffold throughout its degradation. This method is preferable to other functionalization methods that add factors such as hydroxyapatite and BMPs to the scaffold surface,^23^ which can easily be exhausted.

## CONCLUSIONS

We describe an electrospun PCL scaffold blended with naturally occurring lecithin that enhances scaffold biocompatibility, mechanical properties, cell‐seeding efficiency, and osteoinduction. Lecithin acts to reduce scaling of electrospun scaffolds that previously led to large variations in fiber diameters whilst also reducing scaffold hydrophobicity. Reduced fiber curvature also appears to increase scaffold specific elastic modulus, despite a reduction in polymer modulus. Although fiber and pore sizes were reduced through the addition of lecithin, electrospun scaffolds such as these can easily be altered to accommodate many tissue engineering needs such as large surface areas for cellular attachment or larger pores to develop cellular ingression into scaffolds. Moreover, we show how lecithin increases osteoinductive properties of electrospun PCL scaffolds, enhancing osteogenic gene expression. Integration of lecithin throughout the scaffold ensures these beneficial properties are maintained throughout the degradation of the scaffold and not simply isolated to the scaffold surface. Enhanced biocompatibility of such a widely used polymer, PCL, could allow more efficient and tailored scaffolds for use in tissue engineering.

## References

[1] Bassi AK , Gough JE , Zakikhani M , Downes S. The chemical and physical properties of poly(*ɛ*‐caprolactone) scaffolds functionalised with poly(vinyl phosphonic acid‐*co*‐acrylic acid). J Tissue Eng 2011;2011:615328. 22073379 10.4061/2011/615328 PMC3179887

[2] Middleton JC , Tipton AJ. Synthetic biodegradable polymers as orthopedic devices. Biomaterials 2000;21:2335–2346. 11055281 10.1016/s0142-9612(00)00101-0

[3] Li WJ , Tuli R , Huang X , Laquerriere P , Tuan RS. Multilineage differentiation of human mesenchymal stem cells in a three‐dimensional nanofibrous scaffold. Biomaterials 2005;26:5158–5166. 15792543 10.1016/j.biomaterials.2005.01.002

[4] Heo S‐J , Kim S‐E , Wei J , Hyun Y‐T , Yun H‐S , Kim D‐H , Shin J‐W , Shin J-W . Fabrication and characterization of novel nano‐ and micro‐HA/PCL composite scaffolds using a modified rapid prototyping process. J Biomed Mater Res A 2009;89A:108–116. 10.1002/jbm.a.31726 18431758

[5] Gloria A , Causa F , Russo T , Battista E , Della Moglie R , Zeppetelli S , De Santis R , Netti PA , Ambrosio L . Three‐dimensional poly(ɛ‐caprolactone) bioactive scaffolds with controlled structural and surface properties. Biomacromolecules 2012;13:3510–3521. 23030686 10.1021/bm300818y

[6] Sankar D , Shalumon KT , Chennazhi KP , Menon D , Jayakumar R. Surface plasma treatment of poly(caprolactone) micro, nano, and multiscale fibrous scaffolds for enhanced osteoconductivity. Tissue Eng A 2014;20:1689–1702. 10.1089/ten.TEA.2013.0569 24377950

[7] Ko YG , Kim YH , Park KD , Lee HJ , Lee WK , Park HD , Kim SH , Lee GS , Ahn DJ . Immobilization of poly(ethylene glycol) or its sulfonate onto polymer surfaces by ozone oxidation. Biomaterials 2001;22:2115–2123. 11432591 10.1016/s0142-9612(00)00400-2

[8] Alvarez Perez MA , Guarino V , Cirillo V , Ambrosio L. *In vitro* mineralization and bone osteogenesis in poly(ɛ‐caprolactone)/gelatin nanofibers. J Biomed Mater Res A 2012;100:3008–3019. 22700476 10.1002/jbm.a.34233

[9] Gaharwar AK , Mukundan S , Karaca E , Dolatshahi‐Pirouz A , Patel A , Rangarajan K , Mihaila SM , Iviglia G , Zhang H , Khademhosseini A . Nanoclay‐enriched poly(ɛ‐caprolactone) electrospun scaffolds for osteogenic differentiation of human mesenchymal stem cells. Tissue Eng A 2014;20:2088–2101. 10.1089/ten.tea.2013.0281 PMC4137355 24842693

[10] Rutledge GC , Fridrikh SV. Formation of fibers by electrospinning. Adv Drug Deliv Rev 2007;59:1384–1391. 17889398 10.1016/j.addr.2007.04.020

[11] Binulal NS , Deepthy M , Selvamurugan N , Shalumon KT , Suja S , Mony U , et al. Role of nanofibrous poly(caprolactone) scaffolds in human mesenchymal stem cell attachment and spreading for *in vitro* bone tissue engineering–response to osteogenic regulators. Tissue Eng A 2010;16:393–404. 10.1089/ten.TEA.2009.0242 19772455

[12] Badami AS , Kreke MR , Thompson MS , Riffle JS , Goldstein AS. Effect of fiber diameter on spreading, proliferation, and differentiation of osteoblastic cells on electrospun poly(lactic acid) substrates. Biomaterials 2006;27:596–606. 16023716 10.1016/j.biomaterials.2005.05.084

[13] Eichhorn SJ , Sampson WW. Relationships between specific surface area and pore size in electrospun polymer fibre networks. J R Soc Interface 2010;7:641–649. 19812071 10.1098/rsif.2009.0374 PMC2842785

[14] Dodson C. Spatial variability and the theory of sampling in random fibrous networks. J R Stat Soc Ser B (Method) 1971;33:88–94.

[15] Eichhorn SJ , Sampson WW. Statistical geometry of pores and statistics of porous nanofibrous assemblies. J R Soc Interface 2005;2:309–318. 16849188 10.1098/rsif.2005.0039 PMC1578270

[16] Livak KJ , Schmittgen TD. Analysis of relative gene expression data using real‐time quantitative PCR and the 2(‐Delta Delta C(T)) method. Methods (San Diego, Calif) 2001;25:402–408. 10.1006/meth.2001.1262 11846609

[17] Zhu Y , Gao C , Shen J. Surface modification of polycaprolactone with poly(methacrylic acid) and gelatin covalent immobilization for promoting its cytocompatibility. Biomaterials 2002;23:4889–4895. 12361630 10.1016/s0142-9612(02)00247-8

[18] Yang F , Wolke JGC , Jansen JA. Biomimetic calcium phosphate coating on electrospun poly(ɛ‐caprolactone) scaffolds for bone tissue engineering. Chem Eng J 2008;137:154–161.

[19] Christopherson GT , Song H , Mao HQ. The influence of fiber diameter of electrospun substrates on neural stem cell differentiation and proliferation. Biomaterials 2009;30:556–564. 18977025 10.1016/j.biomaterials.2008.10.004

[20] Oh S , Brammer KS , Li YSJ , Teng D , Engler AJ , Chien S , et al. Stem cell fate dictated solely by altered nanotube dimension. Proc Natl Acad Sci USA 2009;106:2130–2135. 19179282 10.1073/pnas.0813200106 PMC2650120

[21] Murphy CM , Haugh MG , O'Brien FJ. The effect of mean pore size on cell attachment, proliferation and migration in collagen–glycosaminoglycan scaffolds for bone tissue engineering. Biomaterials 2010;31:461–466. 19819008 10.1016/j.biomaterials.2009.09.063

[22] Engler AJ , Sen S , Sweeney HL , Discher DE. Matrix elasticity directs stem cell lineage specification. Cell 2006;126:677–689. 16923388 10.1016/j.cell.2006.06.044

[23] Seyedjafari E , Soleimani M , Ghaemi N , Shabani I. Nanohydroxyapatite‐coated electrospun poly(l‐lactide) nanofibers enhance osteogenic differentiation of stem cells and induce ectopic bone formation. Biomacromolecules 2010;11:3118–3125. 20925348 10.1021/bm1009238

